# Hydrogenated TiO_2_ Thin Film for Accelerating Electron Transport in Highly Efficient Planar Perovskite Solar Cells

**DOI:** 10.1002/advs.201700008

**Published:** 2017-05-16

**Authors:** Xin Yao, Junhui Liang, Yuelong Li, Jingshan Luo, Biao Shi, Changchun Wei, Dekun Zhang, Baozhang Li, Yi Ding, Ying Zhao, Xiaodan Zhang

**Affiliations:** ^1^ Institute of Photoelectronic Thin Film Devices and Technology of Nankai University Tianjin 300071 P. R. China; ^2^ Key Laboratory of Photoelectronic Thin Film Devices and Technology of Tianjin Tianjin 300071 P. R. China; ^3^ Key Laboratory of Optical Information Science and Technology of Ministry of Education Tianjin 300071 P. R. China; ^4^ Collaborative Innovation Center of Chemical Science and Engineering (Tianjin) Tianjin 300072 China; ^5^ Laboratory for Photonics and Interfaces Institution of Chemical Sciences and Engineering School of Basic Sciences Swiss Federal Institute of Technology Lausanne CH‐1015 Switzerland

**Keywords:** electron transport layer, faster electron transport behavior, hydrogen doping, planar perovskite solar cells, room temperature magnetron sputtering process

## Abstract

Intensive studies on low‐temperature deposited electron transport materials have been performed to improve the efficiency of n‐i‐p type planar perovskite solar cells to extend their application on plastic and multijunction device architectures. Here, a TiO_2_ film with enhanced conductivity and tailored band edge is prepared by magnetron sputtering at room temperature by hydrogen doping (HTO), which accelerates the electron extraction from perovskite photoabsorber and reduces charge transfer resistance, resulting in an improved short circuit current density and fill factor. The HTO film with upward shifted Fermi level guarantees a smaller loss on *V*_OC_ and facilitates the growth of high‐quality absorber with much larger grains and more uniform size, leading to devices with negligible hysteresis. In comparison with the pristine TiO_2_ prepared without hydrogen doping, the HTO‐based device exhibits a substantial performance enhancement leading to an efficiency of 19.30% and more stabilized photovoltaic performance maintaining 93% of its initial value after 300 min continuous illumination in the glove box. These properties permit the room‐temperature magnetron sputtered HTO film as a promising electron transport material for flexible and tandem perovskite solar cell in the future.

## 1 Introduction

Lead halide perovskite materials have recently attracted intensive attention because of their unique features, such as high charge carrier mobility,^1^ large absorption coefficient,^2^ direct bandgap, low crystal formation energies,^3^ long diffusion length,^4^ and unique tolerance to structural defects.^5^, ^6^ Efficiencies over 22% have been reported since their first application in solar cells in 2009.^7^ As perovskites possess long charge carrier diffusion lengths and exhibit ambipolar behavior,^8^ variety of perovskite solar cell (PSC) architectures have been developed, such as the mesoporous^9^, ^10^, ^11^ and planar^12^, ^13^, ^14^ devices, n‐i‐p^15^, ^16^, ^17^ and p‐i‐n^18^, ^19^, ^20^ structures. As the planar structure has a simpler fabrication process exempt for the time consumable high temperature treatment of compact electron transport layer and/or mesoporous scaffold, substantial efforts have focused on the n‐i‐p planar PSCs to obtain an exceptional performance with long‐term stability.

As one of the key functional layers in planar PSC devices, the compact electron transport layer (ETL) extracts and transports photogenerated electrons from the perovskite absorber and blocks generated holes transfer to fluorine doped tin oxide (FTO) substrate. A high‐quality ETL, with appropriate conduction and valance band, superior electron mobility, and conductivity is crucial to achieve high efficiency of planar PSCs by reducing excessive charge accumulation and facilitating effective electron injection at the ETL/perovskite interface. TiO_2_ is the most extensively employed electron collector in junction with perovskites. In order to achieve PSCs with excellent performance, high‐temperature sintering is always required to increase its crystallinity for high charge carrier mobility,^21^, ^22^, ^23^, ^24^ which impedes the continuous production and is also incompatible with temperature‐sensitive substrates, such as plastics and substrates with multijunctions.^25^

TiO_2_ films fabricated at low temperature are being ardently pursued,^26^, ^27^, ^28^ and breakthrough progress has been made with the TiO_2_‐based PSCs compatible for flexible substrates. Yang et al. have reported a dense amorphous TiO_2_ film by magnetron sputtering at room temperature, and achieved 15.07% efficiency on flexible substrates.^29^ However, the poor conductivity of amorphous TiO_2_ is an inherent problem, and several methods have been attempted to solve this problem. For example, graphene nanoflakes were used to modify TiO_2_ films processed at 150 °C to provide superior charge collection and improve the photovoltaic performance.^30^ Besides adding conductive substances to improve the conductivity, doping is another crucial strategy commonly used. For example, Y‐doped TiO_2_ sintered at 150 °C was utilized for better electron transport.^31^ Mg‐doped TiO_2_ annealed at 150 °C was introduced to improve electron transfer from perovskite to TiO_2_.^32^ Nevertheless, investigation of room temperature fabricated TiO_2_ is still urgent, which can further simplify the fabrication process and allow the continuous production especially on the cheap plastic substrates such as polyethylene terephthalate.

Hydrogen is usually used as a shallow donor and provides free carriers to enhance the conductivity. While magnetron sputtering is a versatile method to deposit uniform and compact films, ensuring full coverage to prevent shunting and leakage of currents. Herein, we present a room‐temperature prepared amorphous TiO_2_ film with hydrogen doping (HTO) as ETL in planar PSC through magnetron sputtering. The incorporated H provides free carriers and doubles the carrier concentration in TiO_2_ for a higher conductivity. By doping hydrogen, the Fermi level of TiO_2_ film shifts upward as well, leading to higher open‐circuit voltage (*V*_OC_) of PSC. Based on the HTO substrate, planar PSCs eventually obtain an efficiency of 19.30%, significantly higher than the one based on the pristine TiO_2_ film deposited without hydrogen doping (16.58%).

## 2 Results and Discussion

ETL plays a vital role for the performance of PSC as the *V*_OC_ is determined by the difference between the Femi level (*E*_F_) of electron and hole transport layers, and the conductivity of ETL has crucial effect on the series resistance (*R*_S_) of the device, which affects directly the short‐circuit photocurrent density (*J*_SC_) and fill factor (FF). We expect that hydrogen doping will elevate the *E*_F_ of TiO_2_ and increase its carrier concentration for higher conductivity.

**Figure 1:**
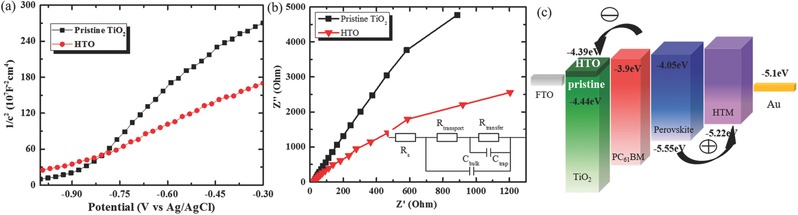
a) Mott–Schottky plots collected at a frequency of 5 kHz for pristine TiO_2_ and HTO films. b) Nyquist plots for EIS measured at 1.6 V versus RHE of pristine TiO_2_ and HTO (the inset diagram is the equivalent circuit model used to fit the Nyquist plots). c) Energy diagram for perovskite solar cells based on pristine TiO_2_ and HTO films.

To verify this hypothesis, electrical properties of pristine TiO_2_ and HTO films are calculated from Mott–Schottky plots, as presented in **Figure** **1**a. Carrier densities and flat band potential of these samples are calculated using the Equations (1) and (2), where *e*_0_ is the electron charge, ε is the dielectric constant of TiO_2_ (=170), ε_0_ is the permittivity of vacuum, *N*_d_ is the donor density, and *V* is the applied bias at the electrode. *C* and *A* are the interfacial capacitance and active area, respectively, *k* is Boltzmann constant, *T* is the absolute temperature, and *e* is the electronic charge

(1) $$N_{d}=\;\;\left(\frac{2}{e_{0}\varepsilon \varepsilon_{0}}\right)\;\;\;{\left[\frac{d(1/C^{2})}{dV}\right]}^{\;-1}$$

(2) $$\frac{1}{C^{2}}=\frac{2}{\varepsilon \varepsilon_{0}A^{2}eN_{d}}\;\left(V-V_{\mathrm{fb}}-\frac{kT}{e}\right)$$

The carrier concentration and flat band potentials calculated from Mott–Schottky plots are shown in **Table** **1**. The carrier concentration of TiO_2_ film increases from 2.25 × 10^21^ to 4.08 × 10^21^ cm^‐3^ with hydrogen doping, indicating we have achieved an amorphous TiO_2_ film with better conductivity, which implies faster electron transport. The flat band potential is slightly lowered with hydrogen doping, implying that the *E*_F_ shifted upward. The incorporated H acts as a shallow donor to provide free carriers, which results in an improved carrier concentration, higher electrical conductivity, and *E*_F_ *_._* In order to confirm that hydrogen doping can improve the conductivity, electrochemical impedance spectra (EIS) analyses were carried out to present the electron transport process in TiO_2_. The Nyquist plots from the EIS measurements are shown in Figure 1b and the fitting results are shown in Table 1. The *R*_transport_ in equivalent circuit model (the inset diagram in Figure 1b, the meaning of other models are presented in Characterization Methods) represents the charge transport resistance in TiO_2_. A significantly reduced *R*_transport_ of HTO indicates better conductivity, which is favorable for better performance of PSCs. The energy levels of PSC devices are presented in Figure 1c, and the energy levels are marked according to the literature.^33^, ^34^ Free charge carriers generated in the perovskite layer can be extracted by either transferring electrons to the underlying PC_61_BM/TiO_2_ layer, or through hole transfer to the Spiro‐OMeTAD hole transport material. As the *V*_OC_ depends on the difference between the *E*_F_ of electron and hole transport layers, PSC based on HTO has a higher *V*_OC_. In addition, the electrons can be more easily injected into the HTO film from the perovskite absorber layer due to the relative small band offset in energy levels between the conduction band of perovskite material and the Fermi level of ETL, implying a smaller loss on *V*_OC_ in electron transport.

**Table 1:** Electrical properties of pristine TiO_2_ and HTO films

TiO_2_	Carrier concentration [10^21^ cm^–3^]	Flat band potential [V vs Ag/AgCl]	*R*_transport_ [Ω cm^2^]
Pristine TiO_2_	2.25	−0.95	1590
HTO	4.08	−1.00	465

**Figure 2:**
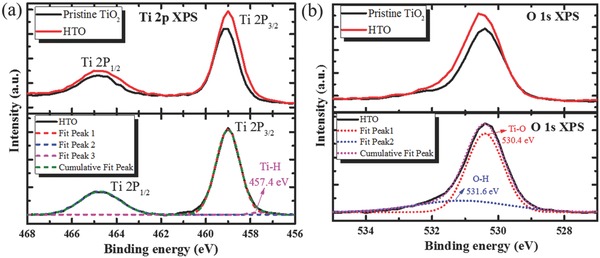
The experimental and fitting results of the pristine TiO_2_ and HTO films: a‐upper) Ti 2P XPS spetra experimental result. a‐lower) The fitting result of HTO film. b‐upper) Normalized O 1s XPS spectra. b‐lower) The fitting result of normalized O 1s XPS spectra from HTO film.

To further clarify the origin of improved carrier concentration and enhanced conductivity by hydrogen doping, the X‐ray photoelectron spectroscopy (XPS) spectra are analyzed to investigate the change of chemical bonding, as depicted in **Figure** **2**. Comparing two samples, the Ti 2p XPS spectra are identical with Ti 2p_3/2_ and 2p_1/2_ peaks centered at binding energies of 458.9 and 464.7 eV, which are typical for the Ti^4+^—O bonds in TiO_2_,^35^ while the additional broad peak centered at 457.4 eV in HTO film is attributed to the Ti—H bonds (H substitution). The Ti—H bonds passivate O vacancies and Ti dangling bonds, efficiently suppressing free charge recombination. In Figure 2b, HTO films exhibit a slight broader O 1s peaks compared to pristine TiO_2_, which can be deconvoluted into two peaks centered at 530.4 and 531.6 eV. The 530.4 eV peak is typical for the Ti—O bonds in TiO_2_, while 531.6 eV peak is attributed to Ti—OH bonds (H interstitial)^36^, ^37^ indicating that hydroxyl is formed through hydrogen doping. The two main bonding ways for H atom incorporated into TiO_2_ films H substitution and H interstitial can both act as shallow donors to increase carrier concentration, which shifts *E*_F_ and improves the electrical conductivity, *J*_SC_ is therefore increased. The influence of hydrogen doping for the morphology of TiO_2_ film is also analyzed through scanning electron microscope (SEM), as shown in Figure S1 in the Supporting Information. HTO film presents a smoother morphology compared with pristine TiO_2_, which is beneficial for the spin‐coating process of subsequent layers.

**Figure 3:**
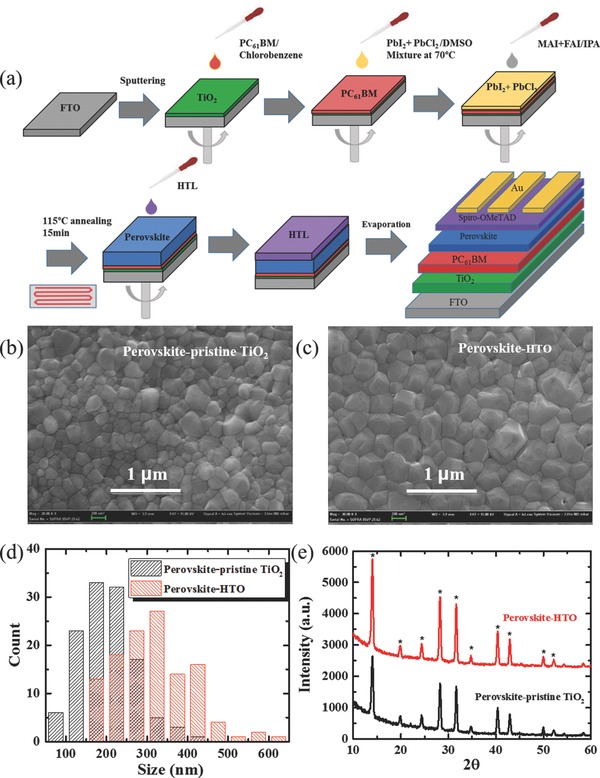
a) Schematic descriptions of device fabrication process. b,c) Top‐view SEM images of perovskite film based on pristine TiO_2_ and HTO, respectively. d) Grain sizes distribution as estimated from the SEM images using Nano measurer 1.2 software. e) X‐ray diffraction spectra of PSCs based on pristine TiO_2_ and HTO. Asterisks denote the perovskite phase.

The pristine TiO_2_ and HTO films are applied to planar PSC devices, whose fabrication processes are schematically depicted in **Figure** **3**a, adapted from a previous report.^38^ PC_61_BM layer is deposited for efficient charge extraction, making the device much less sensitive to the device polarization.^15^ The surface morphology of perovskite films based on pristine TiO_2_ film and HTO is presented in Figure 3b,c, respectively. With the two‐step spin‐coating procedure, the surface exhibits a uniform morphology with dense grains. The entire film is composed of a homogeneous, well‐crystallized perovskite layer, with crystalline grains on the length scale of hundreds of nanometers. The close inspection of the surface images suggests that HTO substrate allows for a larger grain size of perovskite layer (see the statistical distribution in Figure 3d). This may be induced by the smoother morphology of HTO film, which is beneficial for the subsequent spin‐coating process for the PC_61_BM and perovskite layers. The larger grain size and reduced grain boundaries may be beneficial to an increased *J*_SC_. Figure 3e shows the X‐ray diffraction (XRD) spectra of the prepared (FAPbI_3_)_0.48_(MAPbI_3_)_0.52_ films on PC_61_BM/pristine TiO_2_ (HTO)/FTO/glass substrates after annealing at 115 °C for 15 min. The sharp diffraction peaks prove the high crystallinity of the as‐synthesized films, and its characteristic peaks are located at 14.0°, 19.8°, 24.3°, 28.3°, 31.7°, 34.7°, 40.2°, 42.9°, 50.0°, 52.1°, respectively, which is consistent with previously reported data.^9^, ^39^, ^40^ The peak at 14.0°, 28.3°, and 31.7° corresponding to (110), (220), and (310) orientation, respectively are stronger than others, implying a preferential growth direction along these directions. The diffraction peaks of the perovskite film are stronger with the HTO substrate, suggesting better crystalline quality of perovskite.

**Figure 4:**
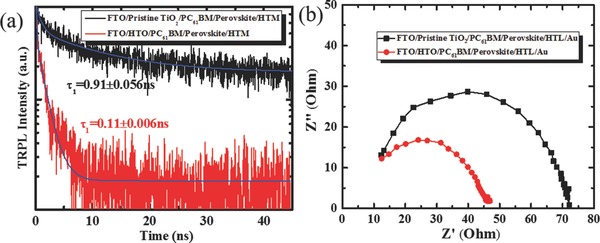
a) Normalized time‐resolved photoluminescence of HTM/Perovskite/PC_61_BM/TiO_2_ deposited with and without hydrogen doping. b) Nyquist plots of PSCs based on pristine TiO_2_ and HTO under 100 mW cm^‐2^ AM 1.5G illumination.

Besides these, the time‐resolved photoluminescence (TRPL) decays are measured to probe the effect of HTO on carrier dynamics in PSCs in **Figure** **4**a. With global biexponential fits, the PL decay of perovskite films based on pristine TiO_2_ and HTO exhibits a time‐constant of τ_1_ = 0.91 and 0.11 ns, respectively, which verifies the faster electron injection rate from perovskite into HTO than pristine TiO_2_, resulting in a higher electron injection quantum efficiency after the electron–hole separation.^41^ We ascribe it to a higher conductivity for HTO film. To further investigate the charge transport process in the devices, Nyquist plots are obtained by measuring alternating current (AC) impedance spectroscopy of the perovskite devices under an illumination of 1 sun without forward bias voltage, as shown in Figure 4b. Impedance spectroscopy measurements reveal the information on the total Ohmic resistance which is associated with the series resistance.^42^, ^43^ The semicircle corresponding to the device based on HTO is smaller than that of pristine TiO_2_ film, which results from the lower internal resistance stemmed from the lower series resistance and better charge injection at the interface between HTO and perovskite material. These data predict the higher FF of the PSC based on HTO.

**Figure 5:**
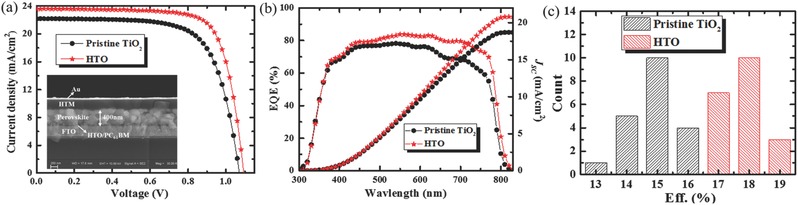
a) The reverse *J*–*V* curves of perovskite solar cells based on pristine TiO_2_ and HTO films measured under simulated AM1.5 sunlight of 100 mW cm^‐2^ irradiance (inset: cross section SEM image of perovskite solar cell based on HTO). b) EQE spectra and integrated J_SC_ for perovskite solar cells based on pristine TiO_2_ and HTO films. c) Statistical histogram of the efficiency values among 20 devices on pristine TiO_2_ and HTO.

**Figure** **5**a,b demonstrates the photocurrent density–voltage (*J–V*), external quantum efficiency (EQE) curves, and the integrated *J*_SC_ of planar PSCs prepared using pristine TiO_2_ and HTO under simulated one sun illumination, respectively. The detail photovoltaic parameters of these devices are given in **Table** **2**. The increased cell efficiency is ascribed to the enhancement of *V*_OC_ *_,_ J*_SC_, and FF values. From a typical cross‐sectional SEM image of a real device, shown in the inset image of Figure 5a, we can see a uniform perovskite layer with 400 nm thickness, which is sufficient for the light absorption. The device exhibits distinct pin‐hole free layers, avoiding the shunting and leakage of currents, which ensures a high *V*_OC_. The upward shifted *E*_F_ of HTO contributes to the *V*_OC_ promotion. The increased FF and *J*_SC_ values are resulted from the reduced series resistance (*R*_S_) due to the high conductivity of HTO and increased carrier concentration with the added donors from hydrogen doping. The accelerated electron injection rate and increased grain size and improved crystallization of perovskite film also contribute to the improved *J*_SC_. Figure 5c illustrates the statistical histogram chart of device calibrated efficiency and the parameters of statistical cells are listed in Tables S1 and S2 in the Supporting Information. It demonstrates that an average efficiency of 15.48% and 18.18% can be achieved respectively for devices fabricated with pristine TiO_2_ and HTO.

**Table 2:** Parameters of perovskite solar cells based on pristine TiO_2_ and HTO films

TiO_2_	Scanning direction	*J*_SC_ [mA cm^–2^]	*V*_OC_ [V]	FF	Eff. [%]	*R*_S_ [Ω cm^2^]
Pristine TiO_2_	Reverse	22.19	1.07	0.70	16.58	5.35
	Forward	21.82	1.07	0.67	15.63	5.92
HTO	Reverse	23.60	1.09	0.75	19.30	4.16
	Forward	23.51	1.09	0.75	19.22	4.06

**Figure 6:**
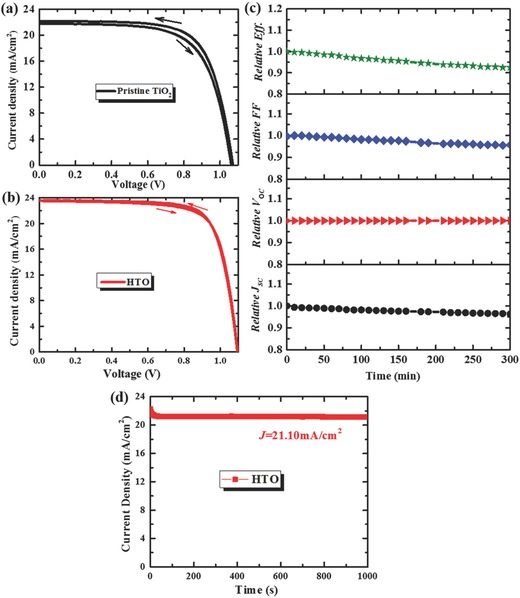
a) *J–V* curves including reverse and forward scan directions of PSCs based on pristine TiO_2_. b) *J–V* curves including reverse and forward scan directions of PSCs based on HTO. c) Normalized parameters of PSCs based on the HTO substrate stored in glove box at room temperature without encapsulation under 100 mW cm^‐2^ AM 1.5G illumination. d) Steady‐state measurement of the photocurrent near the maximum power point at 0.9 V under 100 mW cm^‐2^ AM 1.5G illumination.

**Figure** **6**a,b presents *J–V* curves of PSCs based on pristine TiO_2_ and HTO obtained from forward scan (from short‐circuit to open‐circuit under the forward bias voltage, hereafter abbreviated as FS) and reverse scan (from open‐circuit to short‐circuit under the forward bias voltage, hereafter abbreviated as RS). PSC based on HTO substrate shows less hysteresis compared with the pristine TiO_2_ substrate. To quantify the hysteresis effect, a modified *J–V* hysteresis index (HI) is defined as Equation (3), where *J*_RS_(*V*_OC_/2) and *J*_FS_(*V*_OC_/2) represent photocurrent density at 50% of *V*_OC_ for the reverse and forward scan, respectively.^44^ The HI decreases from 0.045 to 0.008, which might be originated from the increased grain size of perovskite absorber^45^ and the reduced capacitance at the interface between perovskite and ETL, as seen in Figure 3c and Figure 4b, respectively^46^

(3) $$\mathrm{HI}=\frac{J_{\mathrm{RS}}\left(\frac{V_{\mathrm{OC}}}{2}\right)\;\;-\;J_{\mathrm{FS}}\left(\frac{V_{\mathrm{OC}}}{2}\right)}{J_{\mathrm{RS}}\left(\frac{V_{\mathrm{OC}}}{2}\right)}$$

The stability is another important factor in the performance of PSCs, thus it was also investigated for the PSC based on HTO. Figure 6c shows the changes of various parameters, i.e., the stability of the PSC stored in glove box at room temperature under illumination without encapsulation as a function of time. Here, the conversion efficiency decay is defined by Equation (4), where Eff._0_ and Eff._t_ are the efficiencies at the start and the end, respectively. The perovskite cell exhibits very stable performance, with a ΔEff. of ≈7.47% illumination after 300 min. This result is attributed to the highly crystalline properties of the perovskite absorber.

(4) $$\Delta \mathrm{Eff}\cdot =\frac{{\mathrm{Eff}}_{\cdot 0}-{\mathrm{Eff}}_{\cdot t}}{{\mathrm{Eff}}_{\cdot 0}}$$

To determine the steady‐state efficiency, the device was held at an external bias close to the maximum power point and the current was recorded under one sun illumination. As shown in Figure 6d, a stable photocurrent density of 21.1 mA cm^‐2^ was observed at a bias of 0.90 V, indicating that a steady‐state efficiency of the device is obtained.

## 3 Conclusions

In summary, we report a room‐temperature prepared hydrogen doped amorphous TiO_2_ film with an enhanced conductivity applied as electron transport layer in planar PSCs and obtain an efficiency of 19.30%. The incorporated H leads to an improved carrier concentration, higher electrical conductivity, and higher *E*_F_. The improved electrical conductivity facilitates electron transport in ETL. With an elevated electron extraction rate, a higher *J*_SC_ is therefore obtained. The higher *E*_F_ widens the difference between the *E*_F_ of electron and hole transport layers and shortens the relative band offset in energy levels between the conduction band of perovskite and ETL, resulting in the increase of *V*_OC_. Besides these, the HTO substrate is also beneficial to the crystallization of perovskite films, giving much larger grain size with more uniform size. Moreover, the HTO‐based PSC exhibits negligible hysteresis and more stabilized performance. These results presented here permit the room‐temperature sputtered TiO_2_ film as an excellent candidate of the electron transport layer in flexible and tandem perovskite solar cells.

## 4 Experimental Section

*Device Fabrication*: The FTO substrate was ultrasonically rinsed sequentially in detergent, deionized water, and finally dried under a flow of clean air. TiO_2_ films were deposited on the FTO substrate using pulsed direct‐current magnetron sputtering system. Intrinsic TiO_2_ ceramic target (99.999%) was selected and hydrogen flow rate varied with 0 and 5 sccm. Substrate was kept at room temperature. TiO_2_ film thickness was kept as a constant of 56 nm, and the TiO_2_ films were used as an ETL in photoanode. A PC_61_BM (Sigma‐Aldrich) buffer layer was then spin‐coated above at a speed of 3500 rpm for 30 s.

Deposition of perovskite film was performed using sequential method: 1.6 m PbCl_2_ (Sigma‐Aldrich) and PbI_2_ (Sigma‐Aldrich) (PbCl_2_:PbI_2_ molar ratio 1:1) dissolved in the dimethyl sulfoxide (DMSO) (Tianjin Guangfu Fine Chemical Research Institute) solvent (keeping at 70 °C) were spin casted on PC_61_BM layer at 3000 rpm for 60 s, with the film dried at room temperature. Then, 200 µL of methylammonium iodide (MAI) and formamidinium iodide (FAI) (Shanghai Mater. Win. New Materials Corporation) mixed solution dissolved in isopropanol (20 mg mL^−1^, weight ratio 1:1) was loaded on the prepared film for 2 min before spin‐coating at 3000 rpm for 60 s, and then sample was annealed at 115 °C for 15 min. The thickness of perovskite film is ≈400 nm.

The organic hole transporting layer was prepared by dissolving 80 mg Spiro‐OMeTAD (1 material), 28.5 µL 4‐tert‐Butylpyridine (TBP) (Aladdin Reagents), and 17.5 µL LiTFSI (Aladdin Reagents) solution (520 mg Li‐TFSI in 1 mL acetonitrile) in 1 mL chlorobenzene (Aladdin Reagents), and spin casted on the perovskite film at 6000 rpm for 60 s. Finally, gold electrode was thermally evaporated on the top through a shadow mask. Except for electrode evaporation, all fabrication processes were completed in a nitrogen filled glove box. Fabricated devices were stored under dry conditions without sealing and measured in normal ambient atmosphere with the circumstance temperature of 25 °C.

*Characterization Methods*: Film crystal structure of perovskite was examined by XRD spectra (a Rigaku, ATX‐XRD) with Cu Kα radiation (λ = 0.154 nm) in the 2θ range from 10° to 60°. The morphologies of the solar cells were tested using a SEM (Jeol JSM‐6700F). The TRPL spectroscopy was measured with a PL spectrometer (Edinburgh Instruments, FLS 920), and a pulsed laser with a wavelength and frequency of 635 nm and 1 MHz was employed as the excitation source. A long pass filter of 655 nm was used to filter out the excitation light in the transient PL measurements. The Ti 2p and O 1s core peaks were detected using XPS (PHI5000VersaProbe). EIS of the TiO_2_ films were performed in a three‐electrode configuration immersed in a 1 m NaOH aqueous solutions by a Princeton potentiostat electrochemical workstation (PARSTAT 4000), which contains a Pt counter electrode, an Ag/AgCl reference electrode, and TiO_2_ as work electrode. The equivalent circuit model consists of a series *R*_S_ resistance representing the capacity of charge transport in the FTO substrate and external circuit, *R*_transport_ representing charge transport resistance in TiO_2_, *R*_ct_ representing the charge transfer resistance at the interface between the semiconductor and electrolyte. Herein, we mainly focus on the *R*_transport_. EIS of the perovskite solar cells was performed on a PARSTAT 4000 in AM 1.5G in the frequency ranging from 1 Hz to 1 MHz without a bias.

*Photovoltaic Characterization*: Photocurrent density–voltage (*J*–*V*) curves of solar cells were measured at 25 °C under the AM 1.5G (100 mW cm^−2^) illumination. A metal mask with a window of 0.1 cm^2^ was clipped on light injection side to define the active area of the cell. Unless specified, bias scan from 1.2 to −0.2 V first (reverse scan) and return back (forward scan) with a voltage step of 20 mV and delay time 50 ms. Reverse curve is mainly adopted to evaluate the device performance. The spectral response was taken by an EQE measurement system (QEX10, PV Measurement).

## Conflict of Interest

The authors declare no conflict of interest.

## Supporting information

Supplementary
