# Supplementary material for: Hydrogenated TiO2 Thin Film for Accelerating Electron Transport in Highly Efficient Planar Perovskite Solar Cells
Source: Adv Sci (Weinh). 2017 May 16;4(10):1700008. doi: 10.1002/advs.201700008 (PMC5644234; doi:10.1002/advs.201700008)
Supplement: Supplementary file 1 — Supplementary [file ADVS-4-na-s001.pdf]

Copyright WILEY-VCH Verlag GmbH & Co. KGaA, 69469 Weinheim, Germany, 2017.

ADVANCED SCIENCE
Open Access

Supporting Information

for *Adv. Sci.,* DOI: 10.1002/advs.201700008

Hydrogenated TiO_2 Thin Film for Accelerating Electron Transport in Highly Efficient Planar Perovskite Solar Cells

*Xin Yao, Junhui Liang, Yuelong Li, Jingshan Luo, Biao Shi, Changchun Wei, Dekun Zhang, Baozhang Li, Yi Ding, Ying Zhao, and Xiaodan Zhang**

Supporting Information

Hydrogenated TiO_2 Thin Film for Accelerating Electron Transport in Highly-Efficient Planar Perovskite Solar Cells

Xin Yao,[abcd] *Junhui Liang,*[abc] *Yuelong Li,*[abc] *Jingshan Luo,*[e] *Biao Shi,*[abc] *Changchun Wei,*[abc]

Dekun Zhang,[abc] *Baozhang Li,*[abc] *Yi Ding,*[abc] *Ying Zhao,*[abcd] *Xiaodan Zhang* [abcd]*

*E-mail: xdzhang@nankai.edu.cn (Prof. X. Zhang)

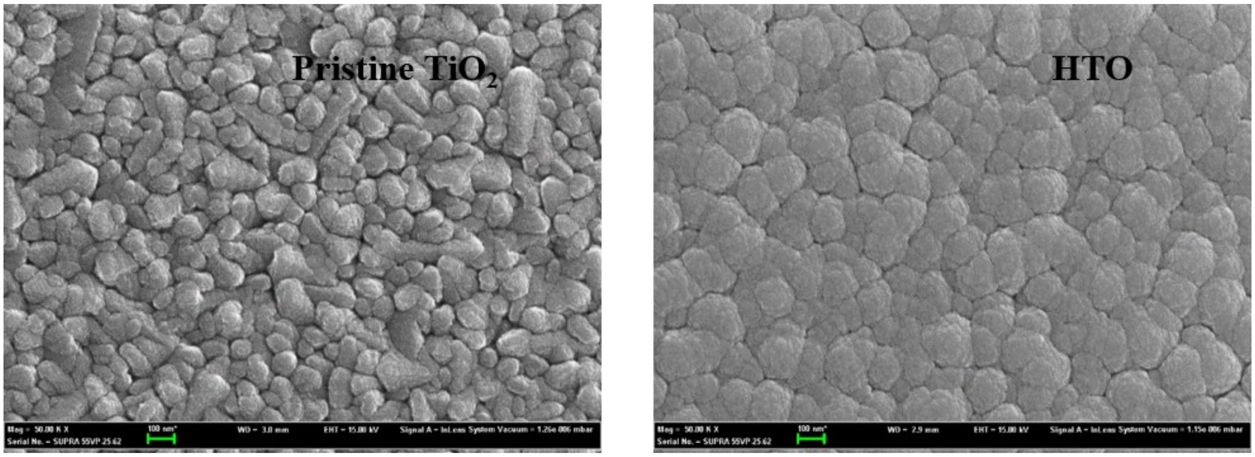

Figure S1 Top-view SEM images of pristine TiO_2 and HTO respectively

Table S1: Statistical data of the efficiency values among 20 perovskite devices based on pristine TiO_2

Samples	Scanning Direction	J_{SC} (mA/cm^2)	V_{OC} (V)	FF	Eff. (%)
1	Reverse scan	20.59	1.07	0.71	15.64
	Forward scan	21.27	1.05	0.64	14.29
2	Reverse scan	21.99	1.07	0.63	14.84
	Forward scan	21.69	1.07	0.59	13.69
3	Reverse scan	22.52	1.05	0.69	16.32
	Forward scan	22.23	1.03	0.63	14.42
4	Reverse scan	21.17	1.07	0.66	14.95
	Forward scan	21.01	1.07	0.59	13.26
5	Reverse scan	21.72	1.07	0.68	15.79
	Forward scan	21.56	1.05	0.66	14.94
6	Reverse scan	21.32	1.07	0.70	15.97
	Forward scan	21.16	1.05	0.65	14.44
7	Reverse scan	21.04	1.07	0.65	14.63
	Forward scan	20.99	1.07	0.60	13.48
8	Reverse scan	20.81	1.07	0.69	15.36
	Forward scan	20.60	1.05	0.63	13.63
9	Reverse scan	20.99	1.07	0.71	15.94
	Forward scan	20.78	1.05	0.64	13.96
10	Reverse scan	20.98	1.07	0.70	15.71
	Forward scan	20.70	1.07	0.62	13.73
11	Reverse scan	20.66	1.07	0.71	15.70
	Forward scan	20.46	1.05	0.64	13.75
12	Reverse scan	20.76	1.07	0.72	15.99
	Forward scan	20.55	1.05	0.66	14.24
13	Reverse scan	21.59	1.07	0.71	16.40

	Forward scan	21.46	1.05	0.65	14.65
14	Reverse scan	22.19	1.07	0.70	16.58
	Forward scan	21.82	1.07	0.67	15.63
15	Reverse scan	21.27	1.05	0.72	16.08
	Forward scan	21.12	1.03	0.63	13.70
16	Reverse scan	21.28	1.07	0.66	15.03
	Forward scan	20.94	1.07	0.67	15.01
17	Reverse scan	20.56	1.05	0.67	14.46
	Forward scan	20.25	1.05	0.64	13.59
18	Reverse scan	20.18	1.05	0.72	15.26
	Forward scan	20.01	1.03	0.67	13.82
19	Reverse scan	21.89	1.05	0.65	14.94
	Forward scan	21.53	1.05	0.59	13.33
20	Reverse scan	20.40	1.07	0.64	13.96
	Forward scan	20.02	1.05	0.62	13.03
Average	Reverse scan	21.20	1.06	0.69	15.48
	Forward scan	21.00	1.05	0.64	14.03

Table S2: Statistical data of the efficiency values among 20 perovskite devices based on HTO

Samples	Scanning Direction	J_{SC} (mA/cm^2)	V_{OC} (V)	FF	Eff. (%)
1	Reverse scan	23.60	1.09	0.75	19.30
	Forward scan	23.51	1.09	0.75	19.22
2	Reverse scan	23.12	1.07	0.73	18.06
	Forward scan	22.99	1.07	0.71	17.47
3	Reverse scan	22.84	1.07	0.73	17.84
	Forward scan	22.73	1.07	0.71	17.27
4	Reverse scan	22.35	1.09	0.74	18.02
	Forward scan	22.29	1.09	0.74	17.98
5	Reverse scan	23.08	1.11	0.74	18.96

	Forward scan	23.04	1.11	0.73	18.67
6	Reverse scan	23.50	1.09	0.75	19.21
	Forward scan	23.51	1.09	0.75	19.21
7	Reverse scan	21.74	1.11	0.73	17.62
	Forward scan	21.65	1.11	0.72	17.30
8	Reverse scan	23.90	1.07	0.75	19.18
	Forward scan	23.79	1.09	0.74	19.18
9	Reverse scan	22.53	1.09	0.75	18.41
	Forward scan	22.46	1.09	0.74	18.11
10	Reverse scan	22.36	1.09	0.74	18.04
	Forward scan	22.25	1.09	0.74	17.95
11	Reverse scan	22.65	1.09	0.73	18.02
	Forward scan	22.52	1.09	0.74	18.16
12	Reverse scan	22.95	1.09	0.72	17.99
	Forward scan	22.81	1.09	0.72	17.94
13	Reverse scan	23.13	1.09	0.72	18.15
	Forward scan	23.02	1.09	0.71	17.83
14	Reverse scan	22.16	1.09	0.74	17.87
	Forward scan	22.06	1.09	0.73	17.55
15	Reverse scan	22.37	1.09	0.75	18.28
	Forward scan	22.31	1.09	0.74	17.99
16	Reverse scan	22.61	1.11	0.73	18.32
	Forward scan	22.52	1.11	0.73	18.25
17	Reverse scan	21.59	1.09	0.75	17.65
	Forward scan	21.51	1.09	0.74	17.35
18	Reverse scan	22.31	1.09	0.71	17.27
	Forward scan	22.21	1.09	0.7	16.95
19	Reverse scan	21.85	1.09	0.72	17.14
	Forward scan	21.67	1.09	0.71	16.77

20	Reverse scan	23.23	1.09	0.72	18.23
	Forward scan	23.13	1.09	0.72	18.15
Average	Reverse scan	22.69	1.09	0.74	18.18
	Forward scan	22.60	1.09	0.73	17.96
